# Ready for action: a role for the human midbrain in responding to infant vocalizations

**DOI:** 10.1093/scan/nst076

**Published:** 2013-06-18

**Authors:** Christine E. Parsons, Katherine S. Young, Morten Joensson, Elvira Brattico, Jonathan A. Hyam, Alan Stein, Alexander L. Green, Tipu Z. Aziz, Morten L. Kringelbach

**Affiliations:** ^1^University Department of Psychiatry, University of Oxford, Oxford, OX3 7JX, UK, ^2^Department of Clinical Medicine, Center of Functionally Integrative Neuroscience, Aarhus University, 8000 Aarhus C, Denmark, ^3^Cognitive Brain Research Unit, Institute of Behavioral Sciences, University of Helsinki and Center of Excellence in Interdisciplinary Music Research, University of Jyväskylä, Finland, and ^4^Department of Neurosurgery, John Radcliffe Hospital, Oxford, OX3 9DU, UK

**Keywords:** periaqueductal gray, infant, parenting, local field potentials, deep brain stimulation, midbrain

## Abstract

Infant vocalizations are among the most biologically salient sounds in the environment and can draw the listener to the infant rapidly in both times of distress and joy. A region of the midbrain, the periaqueductal gray (PAG), has long been implicated in the control of urgent, survival-related behaviours. To test for PAG involvement in the processing of infant vocalizations, we recorded local field potentials from macroelectrodes implanted in this region in four adults who had undergone deep brain stimulation. We found a significant difference occurring as early as 49 ms after hearing a sound in activity recorded from the PAG in response to infant vocalizations compared with constructed control sounds and adult and animal affective vocalizations. This difference was not present in recordings from thalamic electrodes implanted in three of the patients. Time frequency analyses revealed distinct patterns of activity in the PAG for infant vocalisations, constructed control sounds and adult and animal vocalisations. These results suggest that human infant vocalizations can be discriminated from other emotional or acoustically similar sounds early in the auditory pathway. We propose that this specific, rapid activity in response to infant vocalizations may reflect the initiation of a state of heightened alertness necessary to instigate protective caregiving.

## INTRODUCTION

Central to parental care is the adult’s ability to respond to salient infant communicative cues. Vocalizations, such as crying, babbling and laughter, are an infant’s principal means of capturing a caregiver’s attention from a distance. Upon hearing an infant, adults naturally orient towards the source of the sound and make rapid decisions as to whether to approach the infant. Phylogenetically older midbrain regions control such fast, reflexive, approach behaviours. In particular, the periaqueductal gray (PAG) has been ascribed a vast array of functions related to survival and selection of adaptive behavioural responses (Bandler and Shipley, 1994; Linnman *et al.*, 2012), particularly in the context of maternal care (Lonstein and Stern, 1997; Sukikara *et al.*, 2006; Miranda-Paiva *et al.*, 2007; Pavesi *et al.*, 2007). In other animals, pharmacological and lesion studies have indicated a causal role for the PAG in maternal behaviour (for review, see Numan & Woodside, 2010). Although evidence for a similar role in humans is only beginning to emerge (e.g. Noriuchi *et al.*, 2008; Bartels & Zeki, 2004; Laurent & Ablow, 2012), it has been strongly argued that the brain circuits subserving survival functions are conserved across mammals (Ledoux, 2012).

Human vocalizations are the most biologically salient sounds in our environment (Belin, Fecteau & Bédard, 2004). Studies examining the neural representation of vocalizations have consistently implicated midbrain structures such as the inferior colliculus (Holmstrom *et al.*, 2007) and the medial geniculate body (Šuta *et al.*, 2007). The nearby PAG is understood to have an integral role in vocalization production. Stimulation of the PAG elicits vocalizations that resemble natural calls in a variety of species, including rat, guinea pig, cat, squirrel monkey, rhesus monkey, gibbon, chimpanzee and man (e.g. Jürgens, 1994; Vanderhorst *et al.*, 2000). Experimentally induced lesions of the PAG in rodents disrupt the expression of emotional coping reactions to aversive sounds (Bandler, 1988).

Neuroimaging studies of the human PAG have reported increased activity in response to aversive compared with neutral sounds (Zald and Pardo, 2002) and voiced compared to unvoiced speech (Schulz *et al.*, 2005). Activity in the PAG has also been suggested to underlie the pleasurable ‘chill’ experienced when listening to music (Blood and Zatorre, 2001). Although these studies provide indirect evidence for a role for PAG in processing emotionally salient auditory stimuli, they are not informative as to the timing of its involvement.

Given the evidence for PAG involvement in both caregiving behaviour and emotional sound processing, we asked whether the region might have a specific role in responding to human infant vocalizations. To test this, we recorded local field potentials (LFPs) from macroelectrodes implanted in this region in four patients who had undergone deep brain stimulation (DBS). This type of recording allowed us to examine the timing of PAG involvement which has previously been impossible with other human imaging techniques. LFPs were recorded while patients listened to infant vocalizations and artificial control sounds constructed to have a number of the same acoustic properties as the infant vocalizations. Three of the patients also listened to ‘ecological’ control sounds: adult and animal vocalizations. These sounds were chosen because of their similarity to infant vocalizations in terms of their general function in the environment (often eliciting caregiving) and acoustic structure. In addition, LFP recordings were obtained from electrodes implanted in sensory thalamus of these three patients to test the localization of effects.

## MATERIALS AND METHODS

### Participants

Ethical approval of the research methods was obtained from Oxfordshire Research Ethics Committee A (Reference: 08/H0604/58). Participation was voluntary and the patients gave written informed consent to take part in the study.

LFP recordings were obtained from four patients who had undergone DBS surgery for chronic, intractable pain at the John Radcliffe Hospital, Oxford, UK. The first patient was male, aged 31 years and had no children at the time of testing. His pain was related to a brachial plexus injury. The second patient was female, aged 64 years and had two adult children. Her pain was traced back to a leg injury. The third patient was male, aged 30 years and had no children. He had left-sided facial pain. The fourth patient was male, aged 26 years and had no children. His pain was related to a shoulder injury. The three male patients had electrodes implanted in lateral PAG and sensory thalamus and the female patient had electrodes implanted in medial PAG and sensory thalamus.

### Stimuli: infant vocalizations

The infant vocalizations consisted of 45 sound clips with an average duration of 1.39 s (s.d. = 0.17). These stimuli were taken from a larger set of infant sounds, which were rated by an independent sample of 80 adults on measures of infant mood (for a description, see Young *et al.*, 2012). Sounds that were clearly interpreted as ‘cries’, ‘laughs’ and ‘babbles’ based on these ratings were included. These stimuli were obtained from audio recordings from nine infants filmed in their own homes during a play session with their primary caregiver, with the approval of the Oxford Research Ethics Committee. The infants were full-term, healthy and aged between 6 and 8 months (mean = 6.7 months, s.d. = 0.9; five males and four females). All stimuli were free from background noise.

### Constructed control sounds

The constructed control sounds were designed to be physically similar to the infant vocalizations on key dimensions. A frame-by-frame (frame length of 20 ms) analysis was applied to each original infant vocalization and the spectral envelope was extracted. This envelope was then multiplied by a complex sound (*f*_0_ = 500 Hz, with five harmonics). The resulting electrostatic control sounds matched the infant vocalizations for total root-mean-square (RMS) intensity, temporal pattern and overall frequency range, without sounding like an actual infant vocalization. The infant vocalizations and control sounds differed with regards to their exact frequency content. A control stimulus was made to match each individual infant vocalization (45 in total) using MATLAB software (7.9.0.529).

### Ecological control sounds

Ecological control sounds comprised natural sounds: adult and animal distress vocalizations taken from a standardized database (Parsons *et al.*, 2012). In brief, the human adult distress sounds consisted of 15 vocalizations from non-acted sources. Given that there are few opportunities to obtain high-quality recordings of real adult distress vocalizations free from background noise, the adult distress vocalizations were obtained from a number of personal video diaries posted on the Internet. The owner of each diary was contacted and permission was requested for the use of the audio component of the videos for research purposes. This allowed us to obtain natural adult distress vocalizations rather than vocalizations from actors. In total, 15 adult vocalizations were included.

High-quality distress vocalizations of domestic animals were obtained from an Internet source (http://www.freesound.org). A sample of 25 adults rated a larger selection of these sounds on the mood of the adult/animal. Fifteen sound clips from cats and 15 from dogs were included. Ecological control stimuli were on average 1.32 s in duration (s.d. = 0.18) and were free from background noise. These sounds were also matched for total RMS intensity.

### Behavioural task

The patients performed an implicit listening task to ensure attention to the auditory stimuli. Patients were required to press a button on a keyboard when they heard a target pure tone of 400 Hz and ignore a distracter pure tone of 500 Hz presented pseudo-randomly on average once every six experimental stimuli (infant vocalizations and control sounds). Concurrently, LFPs were recorded from between the four contact points of the DBS electrodes. Vocalizations and control sounds (both ecological and artificial) were presented in randomized alternating blocks of 45 stimuli. The interstimulus interval was ∼1 s, but varied up to around 250 ms dependent on the precise length of the individual stimuli. After every two blocks, participants were presented with a progress bar to indicate the remaining duration of the task. The first patient listened to the infant vocalizations and constructed control sounds only and heard each block four times, resulting in a total of 360 stimulus presentations. The three other patients listened to each of these blocks six times, and also to the ecological control sound block (all intermixed) three times, resulting in a total of 675 stimulus presentations.

The experiment was programmed in Presentation software (Version 14.4, NeuroBehavioral Systems, Inc. www.neurobs.com) on a computer running Windows XP (Microsoft, Seattle, WA, USA). This computer was equipped with a parallel output cable that delivered triggers to the LFP recording system at the onset of each auditory stimulus and button press with millisecond precision. The participants listened to the sounds through Sony stereo headphones (MDR-XD200).

### Electrode placements, outcomes and recordings

Following standard procedure at Oxford Functional Neurosurgery, each of the four patients was implanted with two electrodes: one in the periaqueductal/periventricular gray (PAG/PVG) and one in sensory thalamus (Kringelbach *et al.*, 2009). Before surgery, anatomical MRIs were obtained to plan the electrode placement. A stereotactic CT scan was then performed and the pre-operative MRI was combined with the stereotactic CT. The anterior and posterior commissures were identified on the axial images for the localization of electrodes. The electrodes were model 3387 (Medtronic Neurological Division, Minneapolis, MN, USA) with four platinum-iridium cylindrical surfaces (1.27 mm diameter and 2.0 mm exposed tip and 1.5 mm gap between two adjacent electrode contacts). After placing the electrodes in the target locations, the whole stimulation system was connected using extension leads to the pulse generator (IPG—Kinetra TM, Medtronic), which remained externalized for 1 week. Immediately after the operation, each patient had another CT scan to confirm that the electrodes had been implanted in their target locations.

Electrode placement was registered in each patient and transformed to standard MNI space using tools from the FMRIB Software Library (FSL version 4.1, www.fmrib.ox.ac.uk/fsl, University of Oxford). First, all electrode points were identified on the post-operative 1 × 1 × 1 mm CT scan. We then used the FLIRT tool (FSL) to generate a 12-parameter linear transformation from these electrode points by finding the transformation from each individual patient’s post-operative CT to pre-operative MRI scan, and from the pre-op MRI to the standard MNI 1 × 1 × 1 mm. The resulting transformation was applied to the electrode points (using nearest neighbour interpolation) allowing plotting of the coordinates of each electrode’s four contacts in standard MNI space. During the post-operative week, electrode settings were optimized for clinical outcomes. Stimulation typically involved a frequency of 20–40 Hz, pulsewidth of 90–300 ms and 1–5 V.

### LFP recordings

**Fig. 1:**
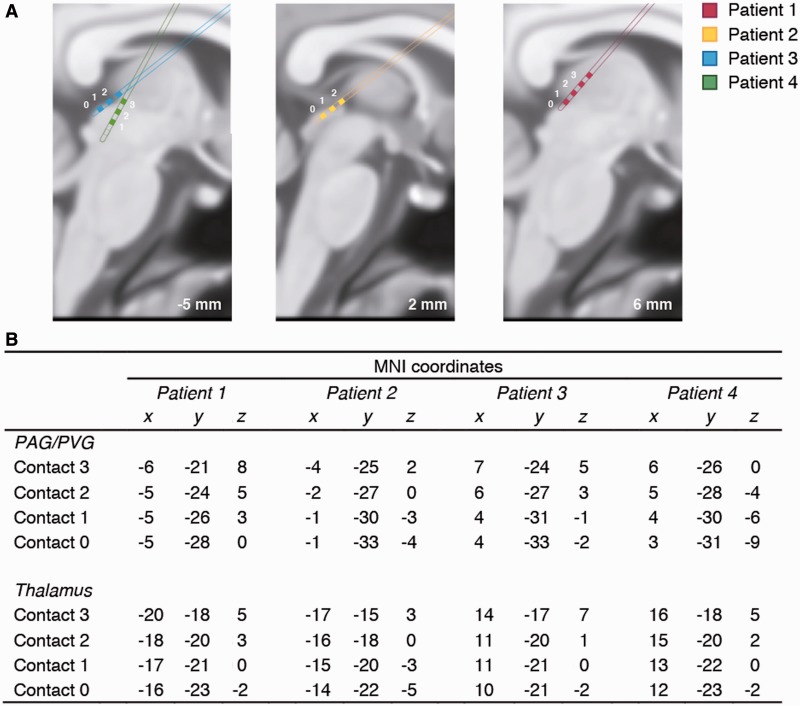
(**A**) Three sagittal slices of the averaged standard brain in MNI space (−5, 2 and 6 mm) showing the approximate locations of the implanted PAG/PVG electrode placements in the each of the four patients (colour coded), with each contact point numbered. Each electrode had four contact points (those points that can be shown in the present slice are in filled colour). (**B**) MNI co-ordinates of the electrode contact points in the PAG/PVG and sensory thalamus. For reference, typical MNI coordinates for the inferior colliculus are (*x*,*y*,*z*) = [6, −33, −9] and for the medial geniculate nucleus (*x*,*y*,*z*) = [17, −24, −2].

LFPs from the PAG/PVG junction (all four patients) and sensory thalamus (three patients) were recorded with a bipolar configuration from the adjacent four circumferential contacts of the DBS macroelectrodes. Raw signals from macroelectrodes were filtered (0.5–500 Hz) and amplified (by a factor of 10 000) using an electrically isolated amplifier (CED 1902, Cambridge Electronic Design Ltd., Cambridge, UK) at a sampling rate of 2480 Hz. We used a data acquisition unit (CED 1401 Mark II) with a measurement range of ±500 µV and a resolution of 0.24 µV. Throughout the recording, LFPs were displayed online and saved onto hard disk using Spike II software® (CED version 5.0). No LFPs were recorded from two of the channels (2–3 in Patient 2 and Patient 4, see Figure 1) due to equipment failures. These were excluded from further analysis. All analysis was carried out using MATLAB (MathWorks Inc., Natick, MA, USA) and Fieldtrip toolboxes (http://fieldtrip.fcdonders.nl; Oostenveld *et al.*, 2011).

### Time-locked LFPs

Line noise (50 Hz and harmonics) was removed using second-order Butterworth notch filters. A high-pass filter of 2 Hz and a low-pass filter of 80 Hz were applied. Filtered data were then baseline corrected using a 200 ms prestimulus period (−200 to 0 ms). Time-locked averages for all categories were calculated across channels for each participant (using the Fieldtrip function ft_timelockanalysis) and converted to standardized values. Outliers falling 3 s.d. outside of the mean amplitude were rejected. Data were then averaged across participants. To quantify differences in voltage between the different stimulus categories across time in each electrode channel, a sample-by-sample one-way analysis of variance (ANOVA) was applied, with condition as the main factor, replicating previous methods (Guthrie and Buchwald, 1991; Blair and Karniski, 1993; Kraskov *et al.*, 2007). This analysis produced a time-resolved significance level for the difference in voltage across conditions (*P* < 0.01). We performed two main comparisons with this method: infant vocalizations/constructed control sounds and infant vocalizations/ecological control sounds. Given the difference in trial numbers in the infant vocalization (*N* = 270) and ecological control sound (*N* = 135) conditions, we also selected a random sample of 135 infant vocalizations and repeated the infant vocalizations/ecological control sound comparison with equal trial numbers.

### Time frequency analysis

Time frequency analysis of the data for each sound category (infant vocalizations, constructed and ecological control sounds) was performed using the multitaper method implemented in Fieldtrip. As with the time-locked analysis, we performed a time frequency analysis of a random subset of the infant sounds (*N* = 135). A sliding window from −200 to 500 ms, in 10 ms steps, was applied to frequencies from 2 to 80 Hz. A relative baseline correction was applied using a pre-stimulus interval of 200 ms. ANOVAs, and *post hoc* Scheffe tests where appropriate, were used to test for differences in the alpha (8–12 Hz), beta (13–20 Hz) and gamma (>30 Hz) frequency ranges between 100 and 200 ms.

## RESULTS

### Behavioural task

Participants made almost no mistakes (<5%) in responding to the target tone in the behavioural task. However, participants were substantially faster at responding during infant vocalizations (mean = 775 ms, s.d. = 307 ms) compared with the control sound blocks (mean = 831 ms, s.d. = 346 ms; *F*(1,90) = 29.71, *P* < 0.01).

### Local field potentials

**Fig. 2:**
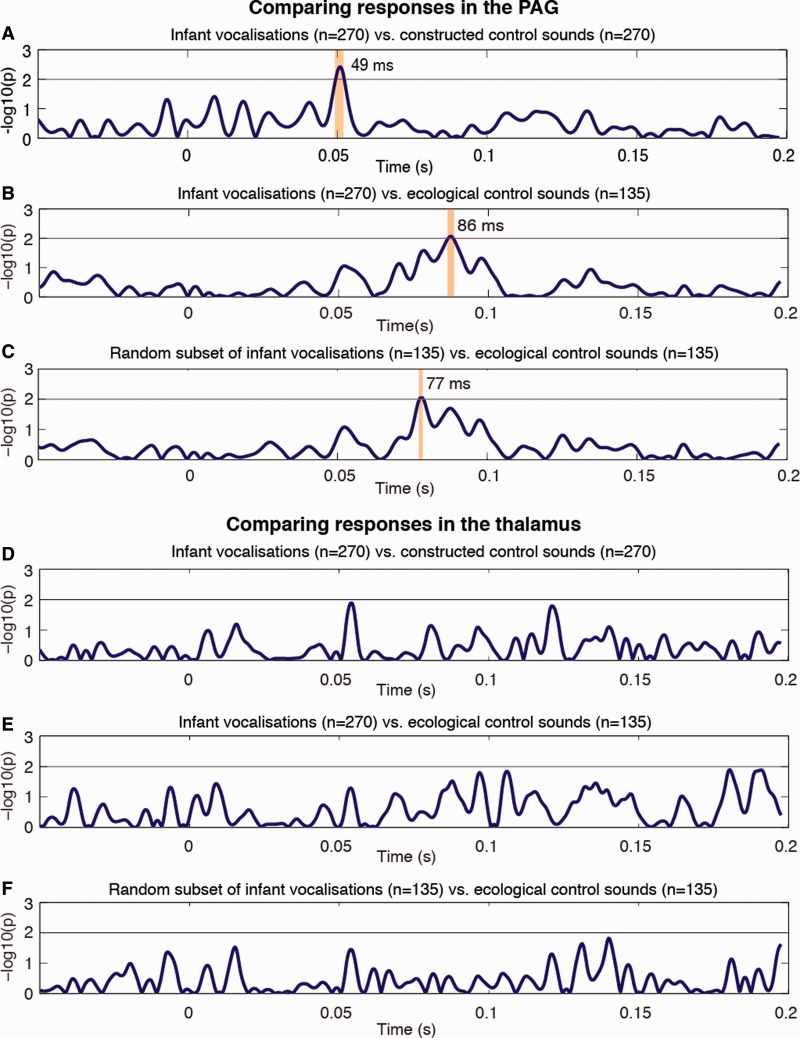
Statistical analysis of the LFP activity in the PAG/PVG and thalamus showing normalized *P*-values obtained from ANOVA tests. (**A–C**) Early differential responses to infant vocalizations sounds in LFPs recorded from the PAG/PVG, presenting (A) all infant vocalizations compared with the constructed control sounds, (B) all infant vocalizations compared with the ecological control sounds and (C) a random subset of the infant vocalizations (*N* = 135) compared with the ecological control sounds (*N* = 135). (**D** and **E**) No differential response to infant vocalizations sounds in LFPs recorded from the sensory thalamus. (D) All infant vocalizations compared with the constructed control sounds, (E) all infant vocalizations compared with the ecological control sounds and (**F**) a random subset of the infant vocalizations (*N* = 135) compared with the ecological control sounds (*N* = 135). Data are presented 50 ms before trial onset (stimulus onset is at time 0 s), to 200 ms after stimulus onset. Significant differences, crossing the alpha 1% threshold, are marked with vertical bars (following the analysis described by Kraskov *et al.*, 2007).

We found a striking early difference at 49 ms in time-locked average LFP activity recorded from the PAG channels in response to infant vocalizations and constructed control sounds (*P* < 0.01, Figure 2A). For the comparison between infant vocalizations and ecological control sounds (animal and adult distress vocalizations), the latency of the first statistically significant difference in time-locked average LFPs was slightly later, occurring at 86 ms (Figure 2B). Comparing LFPs in response to a random subset of the infant vocalizations with those to the ecological control sounds yielded similar results: a significant difference occurred at around 77 ms (Figure 2C).

We also compared averaged LFPs recorded from the thalamus in three patients (nine channels in total, Figure 2D–F). There were no significant differences in these averaged LFPs for any of the comparisons (infant/constructed control sounds; infant/ecological control sounds; random sample of infant sounds/ecological control sound).

**Fig. 3:**
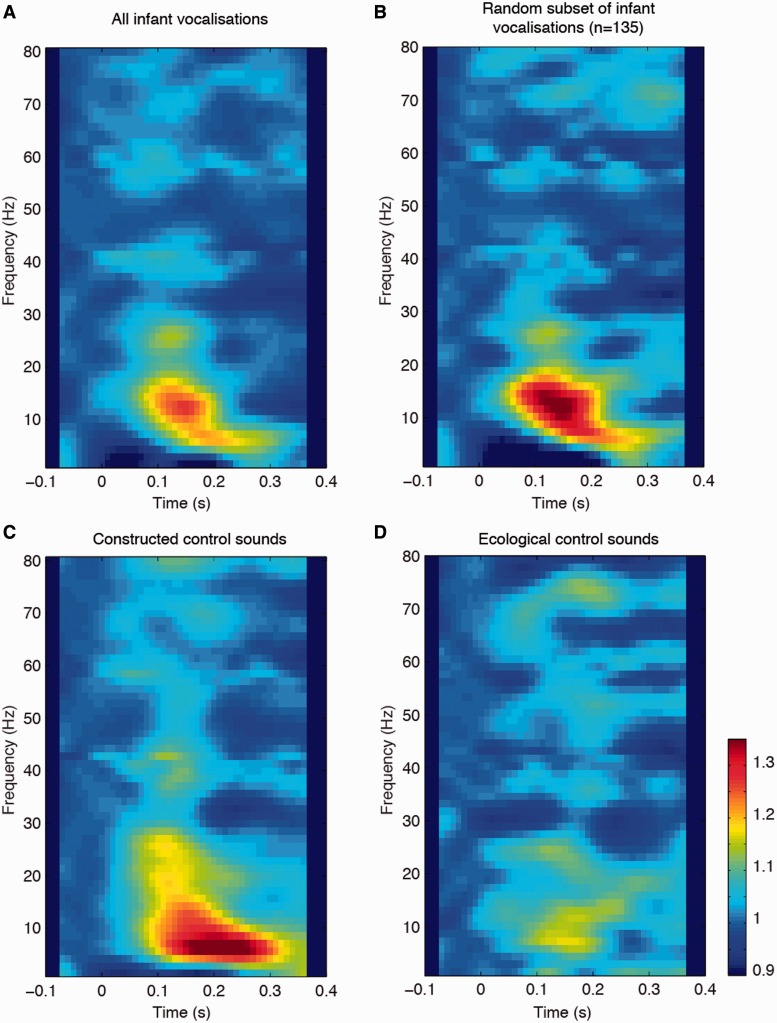
Time frequency analysis of LFPs from the PAG for (**A**) all infant vocalizations, (**B**) a random subset of infant vocalizations (*N* = 135), (**C**) constructed control sounds and (**D**) ecological control sounds. Zero denotes the stimulus onset. Alpha band (8–12 Hz) activity differentiated the animal and adult sounds from the acoustically similar sounds (infant and constructed sounds), whereas beta band (13–30 Hz) activity differentiated the infant sounds from the adult and animal sounds.

Time frequency plots are presented in Figure 3 for the infant vocalizations, a random subset of infant vocalizations (*N* = 135), constructed control sounds and ecological control sounds. From these plots, 100–200 ms post-stimulus presentation was identified as the time window of interest. We found no differences between activity in response to all of the infant vocalizations compared with the random subset of infant vocalizations in any of the frequency bands (*P* > 0.05). There were significant differences in averaged alpha band activity in response to the main three sound categories (*F*(2,674) = 10.07, *P* < 0.0001). This difference was a result of activity in response to the ecological control sounds differing from activity in response to both the infant vocalizations (*P* < 0.0001) and the constructed control sounds (*P* < 0.0001). There was no difference in alpha activity in response to the infant and constructed control sounds (*P* = 0.83). There were also significant differences in averaged beta band activity in response to the different sound categories (*F*(2,674) = 4.45, *P* < 0.01). This difference was a result of activity in response to the infant vocalizations differing from responses to the ecological control sounds (*P* < 0.01), but not constructed control sounds (*P* = 0.71). Activity in response to the constructed control sounds and ecological control sounds was also similar (*P* = 0.07). There were no differences in gamma band activity in response to any of the sound categories.

## DISCUSSION

Here we present the first, direct evidence for early differential processing of infant vocalizations in the human midbrain, recorded from the PAG. These results suggest that human infant vocalizations can be discriminated from other complex sounds with similar acoustic properties, or sounds of high emotional valence, at an earlier stage of the auditory pathway than previously examined. The human PAG may constitute part of the subcortical auditory pathway that initiates rapid responses to biologically salient information. Faster performance on the implicit listening task during the infant sounds further corroborates the notion of a state of heightened alertness and mobility instigated by the PAG. Although midbrain structures such as the inferior colliculus and MGB have been well studied in other animals’ processing of vocalizations, evidence for human PAG involvement, or indeed involvement of other subcortical structures in humans, has been lacking.

Our central finding, a difference between LFPs in response to infant vocalizations and constructed control sounds, was evident early in time in the four patients tested. The onset of the first difference between infant vocalizations and natural control sounds was slightly later overall. One possibility is that natural (infant vocalizations) and matched unnatural sounds (constructed sounds) can be differentiated earlier in time than subcategories of natural sounds. Although the constructed control sounds were matched to the infant vocalizations in terms of their spectral and temporal information, they were quite clearly ‘artificial sounds’. The ecological control sounds, on the other hand, were broadly similar to the infant vocalizations in terms of their initial sound structure and emotive and communicative salience. A tentative suggestion is that discrimination of natural sounds from the same broad functional category (emotional, familiar vocalizations) may be more time-consuming than discrimination of natural from unnatural sounds. However, the current data do not speak directly to such questions.

Our time frequency analyses further supports the idea of differentiation of these sounds in the PAG. Alpha activity was similar in response to the two acoustically similar sound categories, the infant and constructed control sounds, but was distinct for the ecological control sounds. Beta activity, on the other hand, differentiated the infant sounds from the ecological control sounds. These results warrant further investigation because the functional significance of such low-frequency oscillations is still unclear (e.g. Palva and Palva, 2007). Furthermore, there may be differences in the function of oscillations dependent on the brain region in question (e.g. Meltzer *et al.*, 2008) and recordings from human subcortical regions are still necessarily rare.

Given that the exact spatial resolution of the LFP recordings presented here is not known, it could be argued that the differences recorded in the PAG may reflect the firing of large neuronal groups in the nearby inferior colliculus. Recent studies suggest that the spatial spread of LFPs can extend from anywhere between 20 and 400 μm to 6 mm (Wang *et al.*, 2005; Kreiman *et al.*, 2006; Berens *et al.*, 2008; Katzner *et al.*, 2009; Nauhaus *et al.*, 2009; Xing *et al.*, 2009; Kajikawa and Schroeder, 2011) in cortical areas. The electrode recording sites in this study were on average 11 mm from the inferior colliculus. Although contribution from the inferior colliculus to the recorded LFP signals cannot be ruled out, it seems likely that the PAG is the primary source of the differential activity recorded here. Irrespective of the precise spread of our LFP recordings, the overall interpretation of our data remains the same: infant vocalizations are subject to differential processing early in time in the human midbrain. This may represent an important first stage in the processing route that facilitates quick reactions to these biologically salient vocalizations.

There is limited consensus from previous neuroimaging studies as to the spatiotemporal pattern of neural activity underlying the processing of infant vocalizations. Functional MRI studies to date have compared neural activity in response to infant cries *vs* artificial control sounds, generally matched on spectral auditory dimensions. Results highlight a diverse group of brain regions including (i) primary auditory cortex (Seifritz *et al.*, 2003; Sander *et al.*, 2007; Swain *et al.*, 2008; Bos *et al.*, 2010; De Pisapia *et al.*, 2013); (ii) additional temporal lobe regions (STS, MTG and temporal pole Lorberbaum *et al.*, 2002; Seifritz *et al.*, 2003; Bos *et al.*, 2010; Montoya *et al.*, 2012) (iii) other brain areas such as the thalamus (Lorberbaum *et al.*, 2002; Swain *et al.*, 2008), orbitofrontal cortex (Lorberbaum *et al.*, 2002), caudate nucleus, cingulate, insula and fusiform gyrus (Swain *et al.*, 2008).

Relatively little is known about the time course of processing of infant vocalizations. One EEG study demonstrated an increased latency of the N100 ERP component to infant cries compared with a one-syllable word (Purhonen *et al.*, 2001). Early subcortical activity in response to infant cries has also been demonstrated using auditory brainstem recordings (Strait *et al.*, 2009), although the specificity of these responses to infant stimuli has not been established. This study therefore represents an important departure from previous work both in terms of experimental method, using matched ecological and artificial stimuli, and insight into the precise timing of subcortical activity that is specific to infant vocalizations.

In animal studies, representation of species-specific vocalizations by subcortical areas has been widely reported (Šuta *et al.*, 2003, 2007; Portfors *et al.*, 2009). Although we did not perform a direct comparison between LFPs in response to vocalizations from different species, we did compare LFPs in response to infant vocalizations and adult and animal vocalizations (taken as one category). The difference in LFPs in response to infant vocalizations compared with the other vocalization subtypes suggests that there may be species-specific processing of vocalizations in the human midbrain (as seen in other animals) or processing specific to infant vocalizations.

Our findings point to a role for the human PAG in the fast discrimination of infant vocalizations from other functionally and acoustically similar sounds. Neuroanatomical evidence suggests that the PAG is uniquely placed to initiate quick responses to auditory information. The PAG receives input from a constellation of sensory cortical and subcortical regions, including non-primary auditory areas (e.g. STS), superior and inferior colliculi, lateral lemniscus and the nucleus gracilis (Dujardin and Jürgens, 2005). Auditory information may be relayed to the PAG via the inferior colliculus (Huffman and Henson, 1990) or the lateral lemniscus (Sakamoto *et al.*, 1996).

Early midbrain involvement in processing infant vocalizations could be part of a general neural signature for protective caregiving responses in adults. Recent neuroimaging studies have suggested that salient visual cues from the infant may be subject to specialized processing, compared with the same cues from adults (Kringelbach *et al.*, 2008; Parsons *et al.*, 2013). Infant faces have been shown to elicit activity in reward-related regions such as the orbitofrontal cortex, presumably reflecting positive, affective processing. Infant auditory cues differ from infant visual cues in their ability to alert an otherwise non-attentive adult quickly. It would seem reasonable to suggest that infant cues are ‘special’, but the neural processing associated with specific visual and auditory cues may be dependent on their functional role.

To our knowledge, this is the first study to explore the processing of infant vocalizations in humans using invasive recordings. Direct experimental evidence of this is kind is rare, and the PAG is notoriously difficult to study with other methods (e.g. fMRI, EEG and MEG). The number of patients tested was therefore small and the current findings will need replication. As a result of the small numbers, it was not feasible to conduct further comparisons, such as exploring differences in processing across species. Future studies may look to investigate such comparisons, and also how and when infant vocalizations of different valence are discriminated in the auditory brain circuits.

Our novel findings are consistent with the notion that infant vocalizations are a privileged category of emotional vocalization, demanding a quick response from the (male or female) listener. Infant vocalizations uniquely draw the listener to the infant, thereby promoting proximity and interaction. We propose that the early, specific, activity in the PAG in response to infant vocalizations may reflect a ‘high-alert’ state, that is elicited upon hearing an infant’s auditory communication.

Neuroendocrinal studies tell a similar story (for a review, see Parsons *et al.*, 2010). In fathers, heightened testosterone responses to infant vocalizations compared with control sounds (Fleming *et al.*, 2002) suggest an action preparatory response to the infant. In mothers, cortisol elevations and changes in heart rate in response to vocalizations have been found (Giardino *et al.*, 2008), possibly reflecting a comparable readiness for action. A distressed infant’s cry has been shown to elicit autonomic arousal in the listener as indexed by heart rate, hand grip force, blood pressure and skin conductance (Boukydis and Burgess, 1982; Zeskind and Collins, 1987; Bakermans-Kranenburg *et al.*, 2012; Del Vecchio *et al.*, 2009; Out *et al.*, 2010). These physiological changes appear to have perceptible consequence for adult behaviour: listening to a crying infant results in immediate improvements in adults’ motor performance (Parsons *et al.*, 2012). We suggest that our findings go some way towards explaining why hearing an infant’s vocalization is highly rousing and difficult to ignore.

## Conflict of Interest

None declared.

## Supplementary Material

Supplementary Data
