# Supplementary material for: Ready for action: a role for the human midbrain in responding to infant vocalizations
Source: Soc Cogn Affect Neurosci. 2013 Jun 18;9(7):977–84. doi: 10.1093/scan/nst076 (PMC4090964; doi:10.1093/scan/nst076)
Supplement: Supplementary Data [file supp_9_7_977__index.html]

Ready for action: a role for the human midbrain in responding to infant vocalizations — Ready for action: a role for the human midbrain in responding to infant vocalizations — Supplementary Data 

# Ready for action: a role for the human midbrain in responding to infant vocalizations
